# Uncertainty in Medical and Dental Students of Pakistan Regarding Their Future Career and Its Major Causes: A Cross-Sectional Study

**DOI:** 10.7759/cureus.50831

**Published:** 2023-12-20

**Authors:** Waseem Sajjad, Muhammad Iqbal, Muhammad Haziq, Aleena Fatima, Iraj Fatima, Wajahat Ullah Ismail, Rahmat Ali, Qayum Ali Shah, Asmi Shaheen

**Affiliations:** 1 Department of Community Medicine and Public Health, Mayo Hospital Lahore, Lahore, PAK; 2 Department of Orthodontics, de'Montmorency College of Dentistry, Lahore, PAK; 3 Department of Internal Medicine, Mayo Hospital Lahore, Lahore, PAK

**Keywords:** causes of uncertainty, career, dental students, medical students, medical education, uncertainty

## Abstract

Objectives: This study aimed to evaluate the extent of confidence in medical and dental students regarding their careers after being enrolled in their germane institutes and to identify the magnitude of uncertainty in medical and dental students about their careers and the important causes of this uncertainty.

Methods: This study was conducted among enrolled students in different medical and dental colleges and universities of Pakistan, including the public and private sectors, from March 1 to March 15, 2023. The level of confidence in their career was evaluated using a close-ended questionnaire of a three-point Likert scale developed and tested by the investigators and approved by the Ethical Review Board (ERB) along with the synopsis. Data were managed and analyzed using Microsoft Excel 19 (Microsoft Corporation, USA) and IBM SPSS Statistics for Windows, version 27 (released 2020; IBM Corp., Armonk, New York, United States).

Results: This study included 1,126 students from public and private medical and dental institutes. The majority of participants 965(85.7%) were satisfied with their chosen profession, and 1,042 (92.5%) students believed they could make a positive contribution to society. Out of the total participants, 246 (21.8%) students showed their intention of changing careers if provided with a comparable/alternative opportunity. A very small proportion, 154 (13.7%), were dissatisfied with their current clinical training and studies. The study also revealed that extra and unjustified academic pressure from institutions is the leading cause of uncertainty among students. Moreover, the lack of psychological support and counseling provided during the academic years adds to the magnitude of uncertainty.

Conclusion: In Pakistan, a staggering number of medical and dental students are unsure of their future careers and career prospects. The main causes of this uncertainty are the extra, unwarranted academic pressure that institutions place on students and the dearth of psychological support and counseling offered during the course of studies. This study not only highlights the prevailing uncertainty among medical students but also identifies the causes behind it. Addressing these causes can alleviate the prevailing uncertainty and bring about satisfactory and productive academic achievements without suffering from worries about the future.

## Introduction

Uncertainty is the lack of confidence or clarity in one's ideas, decisions, or intentions [1]. The uncertainty not only affects the pace of education but also causes long-lasting pessimistic changes in personality [2]. Medical and dental fields are regarded as among the most rewarding careers in terms of service to humanity, respect, and earnings. For decades, students in Pakistan have regarded these as a top priority. However, the recent, unexpected, and poorly evaluated shoehorning of students into this field has created a remarkable quandary for the enrolled students in terms of their career and job opportunities. In addition, this predicament is exacerbated by market saturation and the subpar accommodations provided by both the public and private sectors.

Above all, poor psychological assessment of students prior to admission and during their studies tenure for optimum performance and optimistic approach development regarding their studies and career has resulted in significant psychological uncertainty among the students [3]. A large number of students are dissatisfied with the way their learning and clinical training are tailored to be applied at professional levels. Medical and dental fields are the fields where one not only has to treat the patients but also play a management and leadership role. This uncertainty not only clogs their learning but also causes continuous mental trauma and confusion and makes them vulnerable to the future challenges of their respective fields [4].

With better healthcare facilities being a fundamental human right, this current dilemma demands serious consideration and appropriate action to remove any doubts it may cause in future medical and dental professionals who will serve humanity [5]. Psychological assessment of students prior to enrollment in studies, as well as proper psychological motivation and guidance throughout the course of studies, can play a significant role [6].

This study aimed to decipher the prevailing magnitude of uncertainty in medical and dental students of Pakistan regarding their future careers and to unmask the underlying roots beyond this uncertainty. This will not only highlight the magnitude of uncertainty in medical and dental students regarding their future careers, but it will also help medical and dental educational institutions to safeguard the mental health of their students and will help medical experts and educational policymakers to reconsider their educational and training moulds that may be causing the unnecessary burden of active or passive strain and uncertainty in medical and dental students harnessing their energy and making their efficiency suboptimal.

## Materials and methods

The study was structured as a cross-sectional survey-based investigation, which means that data were collected at a single point in time to provide a snapshot view of the subject of interest. To gather information, a carefully designed Likert scale-based, multiple choice, close-ended questionnaire was utilized. The questionnaire was developed by the investigators. After pre-testing, it was presented before the ERB along with the complete synopsis and written consent for the participants. This questionnaire featured questions with pre-defined answer choices, a format chosen for its efficiency in data collection, management, and subsequent analysis.

The research took place within various esteemed medical and dental colleges of Pakistan, institutions known for their high standards of education and clinical practice. The study was executed over a specific timeframe, from March 1 to March 15, 2023. This timeline was established following the approval of the research synopsis, ensuring a focused and efficient data collection process. A hard copy of the Institutional Review Board (IRB) approval (no. 560 dated 08/02/2023) was obtained from the concerned IRB prior to data collection.

In terms of participant selection, the aim was to include a substantial number of individuals for a robust dataset. All the students enrolled in the medical or dental institutes of Pakistan were included in the inclusion criteria irrespective of the institution, whether public or private. All those students who appeared in their last exams of medical or dental education were considered ineligible and were excluded irrespective of their results, whether declared or not, passed or failed. Moreover, those students whose admissions were in process but were not attending the institutions were also considered ineligible and were excluded. All those students who are from Pakistan but are enrolled in foreign medical and dental institutions were also excluded.

Keeping the large size of the targeted population into consideration, a snowball, non-probability sampling technique was preferred to conserve financial expenditures. It was chosen for its practicality in this context, striking a balance between feasibility and the generation of reliable data. In this way, the study was able to efficiently capture a representative sample of participants within the given time frame and resource constraints. A sample size ranging from 1,000 to 2,000 participants was targeted, with a confidence level set at 95%. Initially, 1,500 participants were recruited to fill the questionnaire, but upon final scrutiny, only 1,126 were considered for the final evaluation who filled their questionnaire appropriately and completely in order to avoid any disparity in the results and to ensure the quality of the study. The participants were provided with both online and in-hand (hard copy) questionnaires along with written consent, depending upon the feasibility of the data collection and the availability of the targeted sample. Finally, the data were managed and analyzed using Microsoft Excel 19 (Microsoft Corporation, USA) and IBM SPSS Statistics for Windows, version 27 (released 2020; IBM Corp., Armonk, New York, United States).

## Results

In this cross-sectional study, 1,126 students were conscripted, out of which 255 (22.6%) were males and 871 (77.4%) were females. There were 867 (77%) students from the public sector 259 (23%) from the private sector. There were 848 (75.3%) students from the Bachelor of Medicine, Bachelor of Surgery (MBBS) and 278 (24.7%) from the Bachelor of Dental Surgery (BDS). There were 93 (8.3%) students from the first year, 297 (26.4%) from the second year, 485 (43.0%) from the third year, 115 (10.2%) from the fourth year, and 136 (12.1%) students from the final year in their respective fields, i.e., MBBS or BDS (as shown in Table 1).

**Table 1:** Demographics of the participants MBBS: Bachelor of Medicine, Bachelor of Surgery; BDS: Bachelor of Dental Surgery

Demographic value			Frequency (n=1126)	Percentage
Field of studies:	MBBS	848		75.31
	BDS	278		24.68
Institute category:	Public	867		76.99
	Private	259		23.00
Gender:	Male	255		22.64
	female	871		77.35
Year of medical or dental education:	First	93		8.25
	Second	297		26.37
	Third	485		43.07
	Fourth	115		10.21
	Fifth	136		12.07

**Figure 1:**
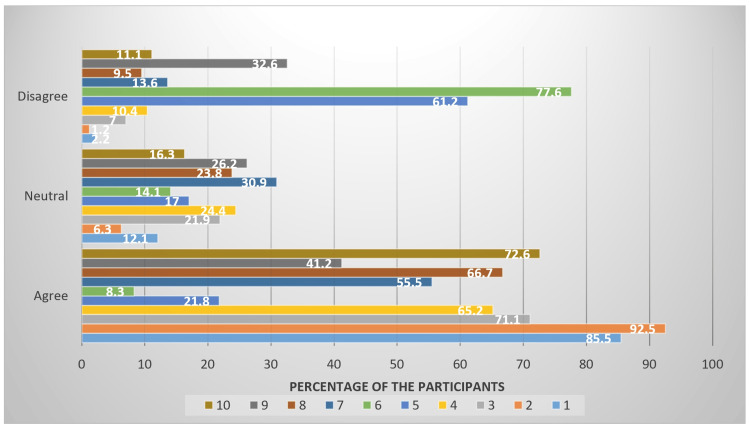
Visual representation of the results. This figure illustrates the percentages of responses of the participants toward different queries in the questionnaire. Queries were as follows: 1. My career choice is a good occupational decision for me. 2. My career will make me able to make significant contributions to the society. 3. The career I am in fits me and reflects my personality. 4. My education and training are being tailored or will be tailored for this field perfectly by the time I graduate. 5. I would like to change my career if provided with a comparable option. 6. All the planning and thoughts I gave for pursuing this career are waste. 7. How much I am satisfied regarding my current studies and clinical training. 8. My field of studies will give me a fruitful career as per my thoughts. 9. I think that the uncertainty I perceive regarding my career is more psychological than reality based. 10. The uncertainty among the students can be decreased through psychological motivation and counseling.

Upon thorough analysis, it was found that only 965 (85.7%) students were satisfied about their career choice, 136 (12.1%) were neutral, while 25 (2.2%) were dissatisfied (see Figure 1 (1)). The number of students that were optimistic regarding their beneficial contributions to society is 1,042 (92.5%), while 71 (6.3%) were neutral and 13 (1.2%) were not confident about it (Figure 1 (2)). According to 801 (71.1%) students, their chosen career is compatible with their personality as compared to 78 (7%) who disagreed while 247 (21.9%) were indifferent (Figure 1 (3)). Out of the total students, only 734 (65.2%) were satisfied with their education and training, 275 (24.4%) were not sure and 117 (10.4%) were dissatisfied about their education and training being tailored perfectly by the time they graduated (Figure 1 (4)). Out of 1,126 (100%), 246 (21.8%) had the tendency to change career if provided with a comparable opportunity while 684 (61.2%) wanted to pursue the same career; the remaining 191 (17%) were ambivalent (Figure 1 (5)). When asked about if the planning and thoughts given for pursuing this career were a waste, 874 (77.6%) students disagreed, 93 (8.3%) thought them to be a waste, and 159 (14.1%) were unsure (Figure 1 (6)). Only 624 (55.5%) students were satisfied with their current studies and clinical training, 348 (30.9%) were neutral, and 154 (13.7%) were not satisfied (Figure 1 (7)). Out of the total subjects, 751 (66.7%) were found to be considering that their chosen field of study will provide them with a fruitful career, 107 (9.5%) were despondent regarding it, and the remaining 268 (23.8%) were neutral (Figure 1 (8)). Among the total, 464 (41.2%) students thought their uncertainty was psychological, 367 (32.6%) considered it reality-based, and 295 (26.2%) were hesitant to declare it as either (Figure 1 (9)). According to 818 (72.6%) students, the uncertainty among the students can be decreased through psychological motivation and counseling, 125 (11.1%) contradicted it, while 183 (16.3%) were indifferent (Figure 1 (10)).

The participants were given six most likely causes, and they were supposed to rank them on the basis of their adding magnitude to the uncertainty. The causes and their ranking on the basis of percentages of responses were in the following manner: The most important cause of the uncertainty was "Extra unjustified academic stress by the institutes leads to psychological stress and unavoidable uncertainty regarding studies and careers" (response of 265 (23.53%) participants). This was followed by "Poor psychological motivation and counseling throughout the course of the study" (response of 241 (21.4%) participants). At number three, the most important cause of uncertainty as per participants was "No/poor psychological assessment of students before admission" (response of 194 (17.22%) participants). After these, the subsequent causes were ranked in the following manner respectively: "Undue fear of examination and high failure ratio create a psychological uncertainty dilemma" (response of 171 (15.18%) participants), "Substandard educational and training facilities" (response of 149 (13.23%) participants), and "Poor communication and understanding between the faculty and the students" (response of 106 (9.41%) participants). 

**Figure 2:**
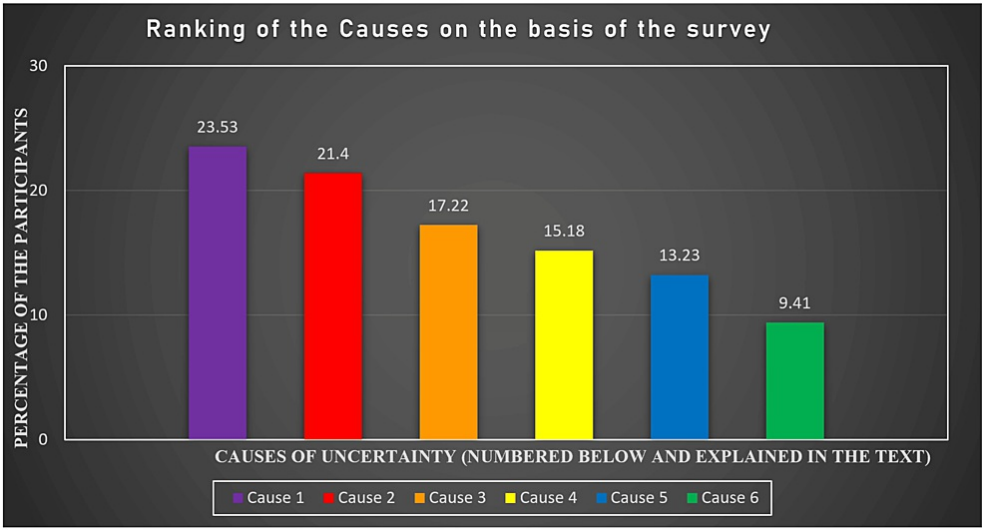
Ranking of the causes of the uncertainty on the basis of responses (colors are devised on the basis of the magnitude of the cause of uncertainty) The values represent the percentages of responses of the participants regarding various causes, as explained in the text. 1. Extra unjustified academic stress by the institutes leads to psychological stress and unavoidable psychological uncertainty regarding studies and career. 2. Poor psychological motivation and counseling throughout the course of studies. 3. No/poor psychological assessment of students before admission. 4. Undue fear of examination and high failure ratio create a psychological uncertainty dilemma. 5. Substandard educational and training facilities. 6. Poor communication and understanding between the faculty and the students. (Note: This list (of causes) is in order corresponding to the ranking established via the responses from the participants.)

All these causes of uncertainty are visually represented in Figure 2, and color has been sought for each, representing the severity and magnitude it adds to the overall perceived uncertainty in the participants. 

## Discussion

This study conducted an assessment of students enrolled in medical and dental colleges and universities across Pakistan, aiming to uncover uncertainties surrounding their career prospects and employment options, as well as the underlying factors contributing to these uncertainties. The results revealed that among the 1,126 students surveyed, a significant majority expressed doubts regarding their future career paths and were uncertain about their bright future. Some felt themselves ill-prepared for their impending professional journeys, while others exhibited a propensity to consider alternative career trajectories if presented with comparable opportunities. These findings collectively underscore a pervasive sense of uncertainty among these students concerning their future professional endeavors and successful careers. This reflects the outcomes of a recently conducted study at Gujranwala Medical College in June 2022, which similarly highlighted pronounced uncertainties within the medical student population of Gujranwala, Pakistan [7].

Moreover, the study unveiled that a substantial number of students displayed a readiness to pivot their career trajectories given suitable alternatives to their current career pathways. This phenomenon is consistent with the recently conducted survey data from India, where a majority of admitted medical students reported uncertainty regarding their specialization, subsequently fostering a predisposition toward career shifts within their chosen field [8]. A recently conducted global survey in France corroborated these findings, linking career indecision and student uncertainty to a high prevalence of depression among the student population [9].

Furthermore, this investigation identified a small yet noteworthy fraction (1.2%) of students who harbored doubts and uncertainty about their potential to make meaningful societal contributions through their future career and work endeavors, while the majority maintained a neutral stance on such apprehensions. In addition, 71 (6.3%) students experiencing heightened confusion were predominantly in their third year of study. This phenomenon may be attributed to the transitional phase from foundational sciences to the clinical realm and applied professional coursework. Another large study conducted at the Memorial University of Newfoundland spanning 2003, 2006, 2007, and 2008 similarly observed heightened career uncertainty among final-year students, influenced by a myriad of factors [10]. These included insufficient psychological assessment of medical school applicants, inadequate educational and training resources, excessive academic pressures, heightened exam-related anxiety, elevated failure rates, and suboptimal teacher-student interactions. The primary objective of the current study was to pinpoint the sources of this ambiguity, ultimately attributing it to excessive and undue stress emanating from educational institutions. 

Another important objective of this study was to unmask the underlying causes of the uncertainty in medical and dental students regarding their future careers. A significant proportion of the participants (23.53%) identified excessive and unjustified academic stress imposed by educational institutions as a major cause of psychological stress and career uncertainty. This aligns perfectly with previous research revealing that medical students are particularly vulnerable to academic stress and test anxiety [11,12]. About 21.4% of the participants pointed out the lack of psychological motivation and counseling throughout their course of study as a cause for their uncertainty [13,14]. The absence or poor implementation of psychological assessments during the admission process was identified by 17.22% of students as a contributing factor. This suggests that understanding the psychological profiles of students could potentially help in providing targeted support and reducing career uncertainty [15]. The fear of examinations and a high failure ratio were reported by 15.18% of students as a cause of career uncertainty. This highlights the need for strategies to manage examination-related anxiety and improve the examination system in medical and dental education [11]. Approximately 13.23% of the participants attributed their career uncertainty to substandard educational and training facilities. This emphasizes the need for standardized and high-quality educational resources and training facilities in medical and dental education [16]. Finally, poor communication and understanding between faculty members and students were reported by 9.41% of the participants as a source of their career uncertainty. Effective communication strategies are crucial in fostering a conducive learning environment [17]. This underscores the importance of providing ample psychological support and counseling to medical students to help them navigate their academic journey and future career paths.

Like any other study, although this study was focused on being ideal, some limitations are still to be acknowledged. There may be variabilities as the study was conducted on a small scale with a small sample size although the actual population of medical students in Pakistan is significantly high. In addition, the questionnaire used was closed-ended, which might not have revealed all the contributing factors. A consecutive non-probability sampling technique was also used, and other techniques might have produced different results. Moreover, owing to the sampling technique and financial constraints, all the medical institutions may not have equal presentation in this study. Equal presentation of all the colleges may reveal somewhat different results.

## Conclusions

In Pakistan, an exceedingly large number of medical and dental students are uncertain about their career and job opportunities. Many thought their field of studies and career path to be incompatible with their personalities. A significant tendency regarding the change of career was seen in medical and dental students, and a large number of students were not satisfied with their study and training, which made them uncertain regarding a bright future and significant contributions to the society. The major factors responsible for this dubiety are the extra unjustified academic stress by institutes, lack of psychological motivation and counseling throughout the course of studies, barriers of communication between the teaching and student bodies, and poor evaluation of the students prior to their enrollment into medical and dental institutions.
